# The feasibility and acceptability of a home-based, virtual exercise intervention for older patients with hepatocellular carcinoma: protocol for a non-randomised feasibility study (TELEX-Liver Cancer)

**DOI:** 10.1186/s40814-022-01069-1

**Published:** 2022-05-27

**Authors:** Samuel T. Orange, Kate Hallsworth, Morven C. Brown, Helen L. Reeves

**Affiliations:** 1 Newcastle University Centre for Cancer, Newcastle University, Newcastle upon Tyne, UK; 2 School of Biomedical, Nutritional and Sport Sciences, Faculty of Medical Sciences, Newcastle University, Newcastle upon Tyne, UK; 3 Population Health Sciences Institute, Faculty of Medical Sciences, Newcastle University, Newcastle upon Tyne, UK; 4 Translational and Clinical Research Institute, Newcastle University, Newcastle upon Tyne, UK; 5 The Liver Unit, Newcastle upon Tyne NHS Foundation Trust, Newcastle upon Tyne, UK

**Keywords:** Liver cancer, Exercise intervention, Telehealth, Quality of life, Feasibility

## Abstract

**Background:**

The number of incident cases and deaths from primary liver cancer, predominantly hepatocellular carcinoma (HCC), has increased markedly in the last two decades. HCC is generally diagnosed at an advanced stage, and most new cases are in people aged over 70 years with age-related comorbidities. Treatment options are often limited, with most patients receiving palliative treatment or supportive care only. As a consequence, maintaining quality of life (QoL) through symptom management is critically important and is a core objective of clinical care. Strong evidence supports the efficacy of supervised exercise training for addressing certain cancer-related symptoms, including QoL, physical function, and fatigue. However, there are many barriers to implementing supervised exercise programmes within cancer care pathways, including economic pressures on healthcare systems and personal barriers for patients. Recent advances in technology allow patients to exercise at home under the ‘virtual’ supervision of an exercise professional through videoconferencing software (termed ‘telehealth exercise’). Despite its potential, there are uncertainties relating to the feasibility, acceptability, and safety of telehealth exercise in people living with HCC.

**Methods:**

This is a protocol for a prospective, single-centre, single-arm, pretest-posttest feasibility trial. We aim to recruit 20 patients aged 60 years or older who have received treatment for HCC and are undergoing routine clinical monitoring. Patients will be invited to take part in two online, home-based, group exercise sessions per week for 10 consecutive weeks. The ‘virtual’ exercise sessions will be delivered in real time by an exercise professional through videoconferencing software. Each session will comprise 30 min of aerobic and resistance exercise performed at a moderate intensity, as guided by the 10-point Borg rating of perceived exertion scale. Feasibility outcomes include recruitment, retention, adherence, intervention fidelity, and safety. Acceptability of the intervention will be assessed using a mixed-methods approach via monthly online surveys and an exit telephone interview. Physical function, accelerometry-measured physical activity, mid-upper arm circumference, and patient-reported outcome measures (PROMS) will be assessed before and after the intervention to determine the feasibility of assessing outcome measures. Physical function outcomes include the short physical performance battery and Liver Frailty Index. PROMS include the Functional Assessment of Cancer Therapy-Hepatobiliary questionnaire, Functional Assessment of Chronic Illness Therapy-Fatigue questionnaire, Activities-specific Balance Confidence scale, Hospital Anxiety and Depression Scale, and the Godin Leisure-Time Exercise Questionnaire.

**Discussion:**

This mixed-methods study will address uncertainties relating to the feasibility and acceptability of delivering live, online, home-based, group exercise sessions to patients with HCC. The findings will inform whether any modifications are required to refine and optimise the intervention, and the assessment of outcome measures will provide information on the likely size and variability of intervention effects. Collectively, the data generated will inform the design of a subsequent, adequately powered, randomised controlled trial to evaluate the efficacy of the telehealth exercise intervention.

**Trial registration:**

ISRCTN14411809

**Supplementary Information:**

The online version contains supplementary material available at 10.1186/s40814-022-01069-1.

## Introduction

Primary liver cancer is the seventh most common cancer and third leading cause of cancer death worldwide, accounting for more than 8% of all cancer deaths [1]. Global cases of incident liver cancer and mortality are increasing. The Global Burden of Disease Study reported that from 1990 to 2017, the number of new liver cancer cases and liver cancer-related deaths has doubled [2]. In the United Kingdom (UK), liver cancer has shown the fastest rise in mortality rates for both males and females in the last decade [2]. There is also a strong relationship between liver cancer mortality and deprivation in England, as evidenced by the twofold higher mortality rates in the most deprived areas compared with the least deprived [3].

The most common type of primary liver cancer is hepatocellular carcinoma (HCC). HCC is generally diagnosed at an advanced stage, and most new cases are in people aged over 70 years with age-related comorbidities, such as frailty syndrome and sarcopenia [4, 5]. The majority of HCC patients in the UK are not eligible for curative treatment, and the prognosis of HCC is typically poor, with a median survival of approximately 6 months and 5-year survival rate of less than 15% [6, 7]. HCC is commonly accompanied by undesirable symptoms, including pain and fatigue [8], which have a negative impact on functional status and quality of life (QoL) [9]. As a consequence, maintaining QoL through symptom management is critically important and often a core objective of clinical care for patients with HCC [10].

A growing body of evidence supports structured exercise as an adjunct therapy in cancer care. The American College of Sports Medicine (ACSM) roundtable in 2018 reported that specific doses of exercise training can improve QoL, fatigue, physical function, anxiety, and depressive symptoms in people living with and beyond cancer [11]. The review also concluded that supervised exercise is more effective than strictly unsupervised programmes [11]. However, there are many barriers to successfully implementing supervised exercise programmes within cancer care pathways, including economic pressures on healthcare systems and personal barriers for patients. Inconvenient travel distances and a lack of access to appropriate facilities are key barriers to regular exercise for cancer survivors [12, 13], and most patients prefer home-based exercise [14–17]. Cancer survivors also report wanting more guidance on the type and specific characteristics of exercise (i.e. frequency, intensity, and duration) they can safely undertake [18]. The challenge is to develop effective exercise interventions that are accessible and sustainable and can be delivered across local healthcare providers [16].

Recent advancements in videoconferencing technology allow patients to exercise at home under the ‘virtual’ supervision of an exercise professional, termed ‘telehealth exercise’ [19]. The exercise instructor can guide patients through the exercise in real time, mimicking the delivery of traditional facility-based exercise training without the need for travel or access to facilities. Moreover, virtual exercise sessions can be delivered in a group-based format, which may provide a peer-supportive environment. Thus, there is potential for telehealth exercise to optimise the health-related benefits of exercise through expert supervision whilst also circumventing common barriers to exercise and meeting the preferences of cancer survivors.

Despite the potential of telehealth exercise, there are uncertainties regarding its feasibility and acceptability in people living with and beyond cancer. One recent study reported that delivering live, online, group-based exercise to highly functioning older adults via a videoconferencing platform is safe and feasible, as evidenced by no adverse events and high adherence (90%) and satisfaction rates [20]. Another recent study reported no intervention-related adverse events and reasonable adherence (79%) to an online falls prevention programme delivered via videoconference in older adults with mild cognitive impairment [21]. However, a 2020 rapid review of telehealth exercise interventions for cancer survivors found that no studies have used real-time videoconferencing to support the delivery of home-based exercise [22]. There are several considerations that may limit the uptake of telehealth exercise in patients living with HCC, including the need to consistently adhere to online exercise whilst managing the fluctuating symptoms associated with chronic liver disease. Unaddressed issues relating to acceptability and feasibility of telehealth exercise could undermine an evaluation of intervention efficacy [23]. Therefore, in line with Medical Research Council guidance on developing and evaluating complex interventions [23], this study will assess the feasibility, acceptability, and safety of delivering a 10-week telehealth exercise intervention to older patients with HCC. The findings will inform the design of a subsequent, adequately powered, evaluative randomised controlled trial (RCT).

### Aims and objectives

- To examine the feasibility, acceptability, and safety of the telehealth exercise intervention in older patients with HCC
- To assess the feasibility of assessing physical function and patient-reported outcome measures (PROMS) and glean preliminary evidence for the efficacy of telehealth exercise in older patients with HCC
- To address the key uncertainties relating to intervention feasibility and acceptability to inform the design of a subsequent randomised controlled trial

## Methods

### Study design

**Fig. 1:**
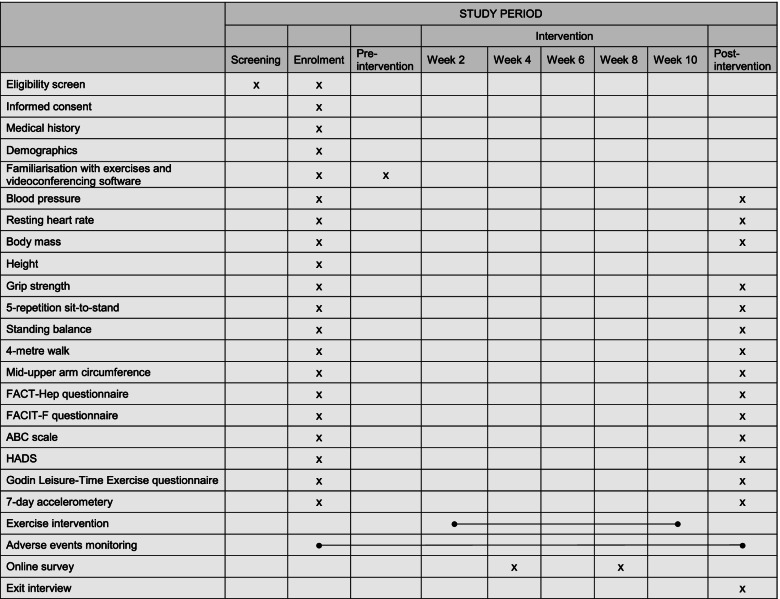
Schematic diagram of the study schedule. FACIT-F, Functional Assessment of Chronic Illness Therapy-Fatigue; FACT-Hep, Functional Assessment of Cancer Therapy-Hepatobiliary; ABC scale, Activities-specific Balance Confidence scale; HADS, Hospital Anxiety and Depression Scale

TELEX-Liver Cancer is a prospective, single-site, single-arm, pretest-posttest feasibility trial. In addition to receiving standard care, patients will receive a 10 week, home-based, virtual, group exercise intervention delivered in real time by an experienced physiotherapist. Feasibility and acceptability of the intervention and outcome measures will be assessed using a mixed-methods approach. A schematic diagram of the study schedule is presented in Fig. 1. The study is prospectively registered on the International Standard Randomised Controlled Trial Number (ISRCTN) registry (ISRCTN14411809). A Standard Protocol Items: Recommendations for Interventional Trials (SPIRIT) checklist [24] is in the supplementary files.

### Study setting

Participants will be recruited from outpatient liver cancer clinics at the Liver Unit at Newcastle upon Tyne Hospitals NHS Foundation Trust (NuTH), UK. It is anticipated that patients will be recruited over a 6-month period. NuTH is the study sponsor. The virtual supervised sessions will be delivered by an NHS-employed physiotherapist, and patients will complete the exercise sessions at home.

### Participant eligibility

#### Inclusion criteria

- Clinical diagnosis of HCC
- Aged 60 years or older
- Have received NHS standard treatment for HCC (determined by stage of disease), with post-treatment imaging reporting a complete response, partial response, or stable disease
- Currently undergoing ‘active monitoring’ at NuTH, involving routine imaging scans and outpatient visits every 3–6 months
- Childs-Pugh of B7 or lower (i.e. preserved liver function)
- World Health Organization performance status of 0 or 1
- Minimum life expectancy of 6 months
- Willing and able to give written informed consent

#### Exclusion criteria

- Aged less than 60 years
- Uncontrolled cardiovascular or metabolic disease
- Breathlessness at rest or with mild exertion
- Severe resting hypertension or (≥ 180/100 mmHg) or tachycardia (≥ 100 bpm)
- Inability to understand written and verbal instructions in English
- Physical disability or mental impairment that precludes safe and adequate participation in the study

### Patient identification and consent process

Patients will be recruited from the Liver Unit at NuTH. After receiving standard of care treatment for HCC, patients typically undergo post-treatment imaging (CT or MRI scan) and attend a follow-up appointment at the specialist liver cancer clinic. The treating physician will identify potential patients ahead of the follow-up appointment by reviewing post-treatment imaging reports and medical records, which are routinely sent for review. A member of the clinical care team will also review the HUNTER trial registry (ISRCTN16680540) to identify potentially eligible patients.

Patients that meet the eligibility criteria will be sent a study invitation letter and participation information sheet by mail. At the clinic visit, the treating physician will discuss the study with eligible patients after they have received standard NHS care (i.e. review, examination, and blood tests). If the patient is interested in taking part, a research team member will provide them with more information at the clinic, answer any questions about the study, obtain written informed consent, and collect baseline data. Baseline data will include medical history, sociodemographic information, body mass, height, resting blood pressure, and resting heart rate.

### Preparation procedures

Patients will be offered an ‘induction’ to the intervention after baseline data is collected. The induction will take place either on the same day as the clinic appointment, in the clinic on an alternative day, or during a home visit, depending on patient preference. Offering home visits is a useful strategy to improve recruitment of patients whom live in rural or non-local areas [25].

During the intervention induction, a member of the research team will show patients how to use the Zoom videoconferencing platform (Zoom Video Communications, California, USA) on a tablet and familiarise them with the exercises and equipment to be used in the study. The researcher will initially demonstrate an exercise and then ask the patient to perform the exercise themselves, with technique adjusted if necessary. Patients will also be instructed on how to self-monitor exercise intensity using the Borg 10-point rating of perceived exertion (RPE) scale [26]. Patients will then receive an intervention pack that includes an instruction manual, exercise diary, RPE scale, incremental level resistance bands (TheraBand, Ohio, USA), pedal exerciser (NRS Healthcare, Leicestershire, UK), and a wrist-worn accelerometer (ActiGraph GT9X Linkm, ActiGraph, LLC., Pensacola, FL). The exercise diary will be used to record daily step counts as well as the type, duration, and intensity of exercise completed each day (including the virtual exercise sessions). Patients without Internet access or an appropriate Internet-enabled device will be provided with a 10.4-inch tablet preloaded with the Zoom app and unlimited 4G data for the duration of the study (Samsung Galaxy Tab A7 10.4” 4G tablet) free of charge. It will be made clear to the patient that they will not be held responsible for loss or accidental damage of any of the equipment supplied by the research team. The equipment will be handed to patients in the clinic or sent by post (via a tracked courier/mailing system) if there are any concerns about the patient’s ability to carry the equipment home.

In addition, the researcher will schedule a 15-min, one-on-one, online introductory session on Zoom with each patient before the intervention begins. During the introductory session to Zoom, the researcher will inspect the location of the exercise area, resolve any technical issues, and ask the patient to practise a sample of the exercises to be used in the study in order to reassess technique and RPE, with exercises modified if required. Patients will be emailed a URL to a password-protected virtual Zoom ‘room’ ahead of their first scheduled exercise session. The same password and URL will be used for all the exercise sessions.

### Exercise intervention

Patients will be invited to take part in two virtual exercise sessions per week for 10 weeks. Exercise sessions will comprise a maximum of 10 patients at any one time to allow for adequate safety monitoring and provision of individual feedback. Sessions will be delivered on weekdays in the late morning or early afternoon, which reflects patient preferences identified in our patient and public involvement (PPI) discussions and also avoids early morning exercise when exercise-induced adverse cardiovascular events are more frequent [27]. Exercise sessions will be separated by at least 48 h.

Exercise sessions will involve the option of chair-based or standing-based exercises (within the same session). Whether patients complete chair-based or standing exercises will depend on patient preference and functional ability (assessed by the research team); patients who take > 15 s to perform five sit to stands at baseline, show an inability to stand for 10 s in side by side/semi-tandem/tandem on baseline balance tests, self-report falling within the last 12 months, and/or score < 50% on the Activities-specific Balance Confidence scale (ABC scale), will be restricted to chair-based exercises in weeks 1–4 because of an increased risk of falls [28, 29]. A fall event is defined as ‘when you land on the floor or the ground, or fall and hit objects like stairs or pieces of furniture, by accident’ [30]. If a patient expresses a preference to change to standing or seated exercises after this period, they will be reassessed virtually and permitted to do so if they meet the requirements stated above. We will ask patients to nominate an emergency contact who we will contact if an adverse event occurs during the exercise sessions. In the case of an emergency, the research team will also contact emergency services and terminate the exercise session for all patients. Furthermore, we will encourage patients to have another person in the house when they are taking part in the scheduled exercise sessions, but if this is not possible, we will ask them to remotely ‘check in’ with their nominated contact after completing each exercise session. Patients will be required to have their cameras turned on and be visible during the exercise sessions so that they can be monitored for potential adverse events.

**Fig. 2:**
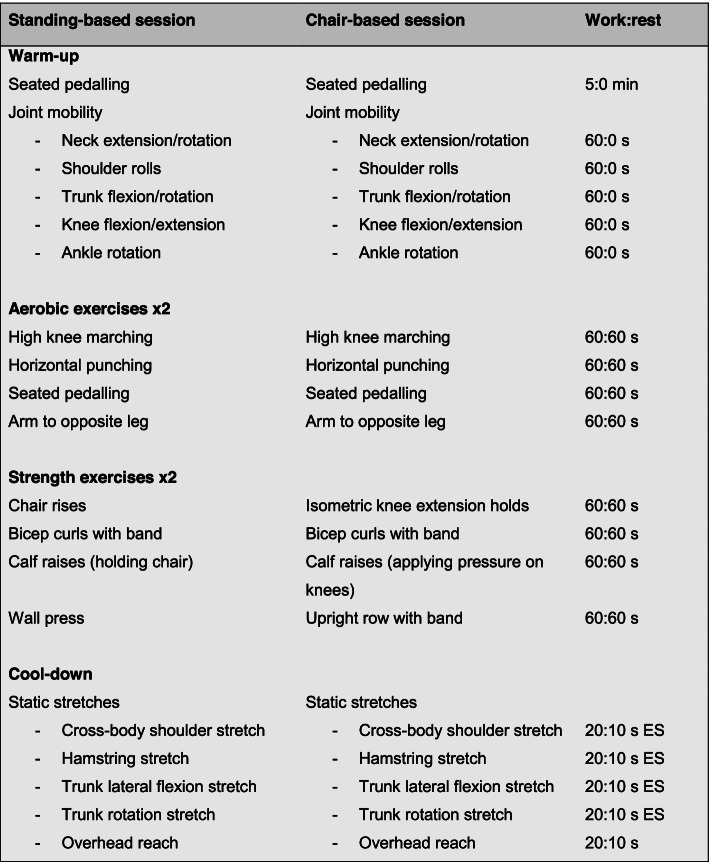
Example of TELEX exercise session. ES, each side

Each exercise session will last 45 min and comprise of a 10 min warm up, 30 min of aerobic and resistance exercises, and a 5 min cool down involving static stretching. The exercises focus on multi-joint movements recruiting major muscle groups in the lower and upper body. The warm up will involve 5 min of a pulse-raising activity (such as seated pedalling) and 5 min of joint mobility exercises. Patients will then perform two sets of four aerobic exercises and two sets of four resistance exercise in a circuit-like manner. Each exercise will be performed for 60 s followed by 60 s of rest; this length of time was chosen to provide an adequate stimulus for adaptation but also provides patients enough time to prepare for the next exercise. Aerobic exercises will include exercises such as seated pedalling, high knee marching, step jacks, side steps, stepping forwards/backwards, and horizontal/vertical punches. Resistance exercises will use body weight and resistance bands as resistance and will include exercises such as chai rises, assisted lunges, horizontal rows, upright rows, overhead presses, wall press-ups, calf raises, rotations, and bicep curls. Each exercise can be modified to be performed in either a seated or standing position. The combination and sequencing of exercises will be varied between the two weekly sessions because our PPI discussions and previous research [20] suggest that older adults taking part in online exercise prefer some level of variation. An example exercise session is presented in Fig. 2.

The intensity of exercise will be performed at 3–6 on the Borg 10-point RPE scale, which corresponds to moderate intensity [31] and qualitative descriptions of ‘moderate’ to ‘hard’ [26]. Moderate-intensity exercise has been shown to improve QoL in cancer survivors [11] and is less likely to trigger acute cardiovascular events compared with vigorous-intensity exercise [32]. The use of RPE to guide intensity ensures that the exercise intervention has inherent progression as participants become accustomed to the exercise. Each session will finish with a cool down of static stretching held for 20 s each at the point of ‘slight discomfort’ [31]. The intervention facilitator will document attendance, adverse events, and any noteworthy technical/practical issues that arise during each session.

### Other intervention components

Patients will have the option of contacting a research team member via telephone or email for assistance if they have any questions or experience any issues, such as technical difficulties using the videoconferencing software. No concomitant care or interventions will be prohibited during the trial [24].

Patients will be able to retain their resistance bands and pedal exerciser at the end of the study to allow them to continue exercising at home after the study has finished. Tablets and accelerometers will be returned to the research team at the follow-up visit to the hospital.

### Outcomes

The primary outcome for this study is feasibility of the exercise intervention and study procedures. Measures of physical function, mid-upper arm circumference, objectively measured physical activity, and PROMs will be assessed before and after the 10-week intervention to determine the feasibility of assessing outcome measures. Acceptability of the intervention will be assessed using a mixed-methods approach via monthly online surveys and an exit telephone interview (see Fig. 1).

#### Feasibility

Feasibility outcomes include recruitment rate, retention rate, intervention adherence, intervention fidelity, and safety. The definition and criteria for success for each feasibility outcome are presented in Table 1. Reasons for declining to participate in the study, and reasons for withdrawing from the study after providing consent, will also be recorded in a Consolidated Standards of Reporting Trials (CONSORT) flowchart.

**Table 1:** Feasibility outcomes for this study

Outcome	Definition
Recruitment rate	The proportion of eligible patients who are approached and give informed consent to participate
Retention rate^a^	The proportion of patients who consent to take part in the study and complete the FACT-Hep questionnaire at both baseline and 10-week follow-up
Intervention adherence^b^	i) The mean proportion of exercise sessions attended by patients (assessed by attendance records kept by the intervention facilitator) ii) The mean proportion of exercise sessions completed at or above a moderate intensity, assessed via RPE (rating of ≥ 3 on the 10-point scale)
Intervention fidelity	Assessed by a research team member attending a 10% sample of the virtual exercise sessions using a standardised checklist to check whether the sessions are delivered in accordance with the protocol
Safety	The number and type (i.e. serious or non-serious) of adverse events that are related or possibly related to the intervention or study procedures

^a^The Functional Assessment of Cancer Therapy-Hepatobiliary (FACT-Hep) questionnaire was chosen for the assessment of retention rate because this is likely to be our primary outcome for a subsequent randomised controlled trial

^b^Adherence to the intervention will be evaluated only in patients who complete the study, defined as patients who complete the FACT-Hep questionnaire at both baseline and 10-week follow-up

#### Physical function

Measures of physical function and mid-upper arm circumference will be assessed during standard of care visits to the outpatient liver cancer clinic at the treating hospital. Physical function will be assessed with the Short Physical Performance Battery (SPPB) and the Liver Frailty Index (LFI). The SPPB is a composite measure of balance, chair stands, and gait speed [33]. Each test is assigned a categorical score from 0 (worst performance) to 4 (best performance) according to standardised criteria, and a total score from 0 to 12 is obtained by summing the scores from the three tests. Higher scores in the SPPB reflect better physical function.

The LFI is a composite measure of hand grip strength, chair stands, and balance [34] and is calculated according to the following formula:

$$LFI=\left(-0.330\times sex\ adjusted\ grip\ strength\right)+\left(-2.529\times number\ of\ chair\ stands\ per\ second\right)+\left(-0.040\times total\ balance\ time\right)+6$$

Grip strength is converted into sex-specific z-scores before being entered into the formula [34]. Total balance time is the sum of the three balance tests in seconds (maximum of 30 s; see below). Lower scores in the LFI reflect better physical function.

##### Hand grip strength

Patients will squeeze a hand-held, analogue, grip dynamometer as hard as possible for 2–3 s using their dominant hand. Patients will remain seated with their arm fully extended by their side throughout the test. The mean score from three trials will be recorded in kg.

##### Five-repetition sit to stand

Patients will begin seated in a firm, armless chair with both arms crossed against their chest. We will ask patients to position themselves on the edge of the chair seat to minimise trunk flexion [35] and instruct them to rise to a full standing position (legs straight) and then return to the seat (full weight on chair) five times, as quickly as they can whilst maintaining correct technique. A practice trial of one repetition will be given to check correct form, followed on by one test trial. The time it takes to complete five sit to stands will be recorded in seconds.

##### Balance

The balance test will involve patients standing unassisted with their feet placed side by side, semi-tandem, and tandem for a maximum of 10 s each. Recording will stop after 10 s or when the patient loses balance (i.e. steps out of position or grabs support). One trial will be performed in each stance position, with the time recorded in seconds.

##### Four-metre gait speed

Patients will walk at their usual pace for 4 m with the time recorded in seconds. Walking aids will be allowed if necessary.

#### Mid-upper arm circumference

The anthropometric measurement of mid-upper arm circumference is a valid measure of muscle mass in nonobese patients [36]. The measurement will be taken with a non-stretching measurement tape at the midpoint between the tip of the acromion and olecranon process of the dominant arm (to the nearest 0.1 cm). Patients will be in a sitting position with the dominant arm hanging relaxed during the measurements [36].

#### Physical activity

Physical activity will be objectively measured using an ActiGraph GT9X accelerometer worn on the nondominant wrist for at least 8 h per day over 7 days in the first and last weeks of the intervention. Daily total activity counts, steps, time spent sedentary, and time spent in light, moderate, and vigorous activity will be recorded. Freedson thresholds based on metabolic equivalents will be used to demarcate to intensity of physical activity: sedentary (≤ 100 counts/min), light (101–1951 counts/min), moderate (1952–5724 counts/min), and vigorous activity (≥ 5725 counts/min) [37, 38].

#### Patient-reported outcome measures

Questionnaires will be given to patients in the clinic to take home and return by post in pre-paid envelopes. The Functional Assessment of Cancer Therapy-Hepatobiliary (FACT-Hep) total score will be used to measure disease-specific quality of life [39]. Fatigue will be measured with the Functional Assessment of Chronic Illness Therapy-Fatigue (FACIT-F) total score [40]. We will use the Activities-specific Balance Confidence scale (ABC scale) total score to assess fear of falls [41]. Anxiety and depression symptoms will be assessed via the Hospital Anxiety and Depression Scale (HADS) total subscale scores for anxiety and depression [42]. Self-reported physical activity will be assessed with the Godin Leisure-Time Exercise Questionnaire using the total leisure activity score [43].

#### Acceptability

We will distribute brief online surveys to patients within 1 h of their second exercise session in weeks 4 and 8 via Google forms. A link to the survey will be distributed via email, and patients will be asked to complete the survey as soon as possible. The survey involves nine Likert-like items on a 5-point scale ranging from 0 (*Strongly disagree*) to 5 (*Strongly agree*), as well as an open-ended section that allows patients to freely express their views. Survey items build on previous research [20] and relate to satisfaction with the technology and exercise sessions. The survey questions are available in the supplementary information files. The research team will meet monthly and decide whether the exercise protocol requires minor modification based on survey findings and feedback during the sessions.

Acceptability will also be assessed qualitatively with in-depth, semi-structured, one-to-one exit interviews with patients. A member of the research team experienced with telephone interviews (MCB), but not involved in intervention delivery, will contact all patients 1–4 weeks after completion of the final follow-up assessment. The interviews will be conducted remotely via telephone. The researcher will facilitate the interviews using a conversational-style approach whilst referring to a topic guide, which is informed by the capability-motivation-opportunity-behaviour (COM-B) model of behaviour [44] and by previous studies exploring experiences and perceptions of exercise [45, 46]. Topics will focus on patients’ perceived expectations, benefits, motives, and barriers to the intervention (see supplementary information for topic guide). The topic guide will be used flexibly to allow patients to raise additional issues which they consider important to the study. It is anticipated that each interview will last approximately 30–60 min.

Final assessments of acceptability will involve examining reasons for declining to participate amongst eligible patients, reasons for non-adherence to the exercise intervention, and reasons for dropout amongst discontinuing patients.

### Safety reporting

Reporting of adverse events will be conducted in line with NuTH’s policy on adverse event reporting for non-clinical trials of investigational medicinal products (CTIMPs). A member of the research team (HLR) will be responsible for determining the attribution and seriousness of adverse events and ensuring they are appropriately documented. All adverse events will be recorded in the Trial Master File. We will report serious adverse events that are deemed to be related to study participation to the trial sponsor and the relevant research ethics committee. Serious adverse events are defined as any untoward medical occurrence that results in death, is life-threatening, requires unplanned or prolonged hospitalisation, results in persistent or significant disability or incapacity, or results in a congenital abnormality/birth defect. Non-serious adverse events are defined as any untoward medical occurrence that do not fulfil any of the serious adverse event criteria [47]. Information on adverse events will be collected after written consent has been contained up until the 10-week follow-up. Patients enrolled into the study are covered by indemnity for negligent harm through NHS schemes. Newcastle University has insurance to cover for nonnegligent harm arising from the design of the research.

### Sample size

There are no clear guidelines on sample size requirements for non-randomised feasibility trials. Thus, our sample size is based on the minimum number of patients the research team considers sufficient to achieve the key aims of the feasibility study and the number that is achievable to recruit within a 6-month period [48]. Based on recruitment to ongoing studies at NuTH with similar participant eligibility criteria (e.g. ISRCTN16680540), we expect that at least three eligible patients per week will be identified. Assuming that 30% of eligible patients provide consent (which is a conservative estimate based on the mean recruitment rate in similar studies [49]), we will recruit at least 20 patients in a 6-month recruitment period. We consider this number of patients sufficient to provide sufficient information on feasibility and acceptability.

### Criteria for success

Based on a systematic review of recruitment, retention, and exercise adherences rates in patients with advanced cancer [49], this feasibility trial will be deemed successful if the following criteria are met:

- ≥ 40% of eligible patients provide written consent to take part in the feasibility trial.
- ≥ 70% of patients attend at least 14 out of 20 exercise sessions.
- ≥ 75% of patients complete the FACT-Hep questionnaire at baseline and 10-week follow-up.
- No serious adverse events are attributable to the intervention or study procedures.

### Data and statistical analysis

#### Quantitative analysis

The flow of patients throughout the trial will be reported in a CONSORT flowchart. Descriptive statistics will be used to present baseline characteristics, feasibility outcomes, and acceptability survey responses. Continuous variables will be described with the mean and standard deviation (SD), and categorical variables will be reported as frequency and proportion. Continuous data with an asymmetrical distribution will be summarised with the median and interquartile range. A paired *t*-test or Wilcoxon signed-rank test (depending on data distribution) will be used to evaluate changes in outcomes from baseline to post-intervention, with the mean difference and 95% confidence interval from the model presented. Data will be analysed per protocol (i.e. missing data at follow-up will not be imputed).

#### Qualitative analysis

Exit interviews will be audio-recorded and transcribed verbatim. Anonymised transcripts will be imported into NVivo qualitative data analysis software (version 12) and analysed using reflexive thematic analysis [50, 51]. This analytic method involves the researcher undertaking six iterative phases of familiarising themselves with the data, generating codes (where we will use an inductive approach), constructing themes, reviewing themes, defining and naming themes, and finally producing the report. All transcripts will be coded independently by one member of the research team, with a proportion (~50%) independently coded by a second team member. Throughout the process, the two researchers will work collaboratively to discuss and refine codes, as well as collating them to develop the potential themes and later reviewing to agree final themes and subthemes.

### Research ethics approval

An independent NHS Research Ethics Committee (Northeast — Newcastle and North Tyneside 2) has ethically approved the study (IRAS ID: 300809).

### Patient and public involvement

We have involved patients and other key stakeholders at various points during the intervention development process. We initially shared an outline of our proposal with the national patient support group: LIVErNORTH. Their members felt that patients with liver cancer would embrace the opportunity to take part in online exercise sessions with other like-minded individuals, particularly because of the COVID-19 pandemic, which has made many people feel even more isolated. They also felt that the exercise sessions may give patients a sense of control over their prognosis. Subsequently, we convened a multidisciplinary steering group that included patient representatives, healthcare professionals (e.g. physiotherapist and hepatologist), and clinical exercise physiologist. The steering group regularly met online to codesign the intervention based on the best available evidence, logistical concerns, safety, and ways to support intervention adherence. We also had in-depth telephone discussions with five patients currently living with HCC (two receiving supportive care and three receiving active treatment). They expressed their views on various aspects of the intervention, and wherever possible, their preferences were incorporated into the research design. Following the codesign process, we held an online focus group with people living with and beyond cancer to gather their feedback on the prototype intervention, which led to minor modifications to the protocol. Patients will continue to be involved in the study via monthly participant surveys as well as through a Patient Reference Group (PRG). The PRG will provide guidance on key issues such as recruitment and dissemination and feed directly into the steering group via the group chair.

### Modification of the protocol

Any modifications to the protocol will be agreed by the research team and trial sponsor and approved by an independent NHS Research Ethics Committee.

## Discussion

Patients with HCC typically face limited treatment options due to age-related morbidities and the advanced stage at which the disease is diagnosed. Most patients are not eligible for curative treatment and are offered palliative treatment or supportive care only [7]. As a consequence, maintaining QoL and day-to-day function through symptom management is critically important [10]. Strong evidence supports the efficacy of specific doses of supervised exercise training for addressing certain cancer-related symptoms, such as QoL, physical function, fatigue, and symptoms of anxiety and depression [11]. However, there are many barriers to implementing supervised exercise programmes within cancer care pathways, including economic pressures on healthcare systems and personal barriers for patients.

Telehealth exercise offers patients the opportunity to take part in virtually supervised group exercise in their own home [19]. This may remove some of the barriers for exercise participation and offers a relatively low-cost means of delivering exercise at scale for a patient population that covers a wide geographical location. Despite its potential, there are uncertainties relating to the feasibility, acceptability, and safety of telehealth exercise in people living with HCC.

This mixed-methods study will address uncertainties relating to the feasibility and acceptability of delivering live, online, home-based exercise to patients with HCC. The findings will inform whether any modifications are required to refine and optimise the intervention, and the assessment of outcomes will provide information on the likely size and variability of intervention effects. Collectively, the data generated will inform the design of a subsequent, adequately powered, randomised controlled trial to evaluate the efficacy of the telehealth exercise intervention.

## Supplementary Information

**Additional file 1: Supplementary information 1.** SPIRIT checklist.

**Additional file 2: Supplementary information 2.** Patient surveys (to be sent monthly via Google Forms).

**Additional file 3: Supplementary information 3.** Exit interview topic guide.

## Data Availability

This manuscript does not contain any data. Supporting material is available as supplementary information files.
