# Supplementary material for: The feasibility and acceptability of a home-based, virtual exercise intervention for older patients with hepatocellular carcinoma: protocol for a non-randomised feasibility study (TELEX-Liver Cancer)
Source: Pilot Feasibility Stud. 2022 May 27;8:113. doi: 10.1186/s40814-022-01069-1 (PMC9135985; doi:10.1186/s40814-022-01069-1)
Supplement: Supplementary file 2 — Additional file 2: Supplementary information 2. Patient surveys (to be sent monthly via Google Forms). [file 40814_2022_1069_MOESM2_ESM.docx]

**Supplementary Information 2**. Patient surveys (to be sent bi-weekly via Google Forms)

|  | Strongly disagree | Disagree | Neutral | Agree | Strongly Agree |
| --- | --- | --- | --- | --- | --- |
| I find “Zoom” is easy to use |  |  |  |  |  |
| I find the exercise equipment easy to use |  |  |  |  |  |
| I can hear the instructor clearly during the exercise sessions |  |  |  |  |  |
| I can see the instructor clearly during the exercise sessions |  |  |  |  |  |
| I would prefer music to be played in the background during the exercise sessions |  |  |  |  |  |
| The 1-10 rating of perceived exertion scale is easy to use |  |  |  |  |  |
| I find the exercise sessions easy to follow |  |  |  |  |  |
| The variety of exercises is right for me |  |  |  |  |  |
| The difficulty of the exercise is right for me |  |  |  |  |  |
| Please make any additional comments |  | | | | |
