# Supplementary material for: The feasibility and acceptability of a home-based, virtual exercise intervention for older patients with hepatocellular carcinoma: protocol for a non-randomised feasibility study (TELEX-Liver Cancer)
Source: Pilot Feasibility Stud. 2022 May 27;8:113. doi: 10.1186/s40814-022-01069-1 (PMC9135985; doi:10.1186/s40814-022-01069-1)
Supplement: Supplementary file 3 — Additional file 3: Supplementary information 3. Exit interview topic guide. [file 40814_2022_1069_MOESM3_ESM.docx]

**Supplementary Information 3**. Exit interview topic guide

1. Before joining the programme, what did you expect from it?
2. How does what you received from the programme differ from what you have expected?
3. What did you want to achieve from the programme? What have been your main motivations for taking part? [Prompts: fitness; function; general health; social contact; something else?]
4. What has been your experience of the exercise programme?

- Were there any parts of the programme that you particularly enjoyed or found the most useful?
- Are there parts of the programme that were particularly onerous or that you would have liked to have been done differently?

1. Are there any health benefits you feel that you have gained after taking part in the exercise programme? How successful do you believe the programme has been for you? [Prompts: fitness/function; mood/energy levels; use of tools within the programme; support provided from the programme]
2. Have you noticed any changes in the way you think about exercise over the course of the programme?
3. Do you plan to continue exercising beyond the study? Why [not]?

- Are there any obstacles that would prevent you from being physically active in the future?
- Do you have any tips that you could give to others for sticking to the exercise programme?

1. Would you recommend this type of exercise to other people living with cancer? Why [not]?
2. Has anything in your life changed since you started the programme? [Prompts: change in circumstances that has positively or negatively affected your progress; physiological or psychological changes?]
3. How useful has the programme been for changing your lifestyle long-term?
4. Is there anything else you would like to add?
